# Symptom and Functional Clusters Regarding Patient-Reported Outcome Measures and Associated Factors Among Patients with Stroke: A Multicenter Cross-Sectional Study

**DOI:** 10.3390/healthcare14152289

**Published:** 2026-07-28

**Authors:** Yanjin Huang, Chaoyue Xu, Qi Wang, Qin Ma, Yan Yue, Xi Chen, Changrong Yuan

**Affiliations:** 1 School of Nursing, Fudan University, 305 Fengling Rd., Shanghai 200032, China; huangyanjin@fudan.edu.cn; 2 School of Nursing, Ningxia Medical University, No. 1160, Shengli Street, Xingqing District, Yinchuan 750004, China; maqin@nxmu.edu.cn (Q.M.); yueyan@nxmu.edu.cn (Y.Y.); 3 Sun Yat-Sen Memorial Hospital, Sun Yat-Sen University, 107 Yanjiang West Road, Yuexiu District, Guangzhou 510120, China; 20222015111461@stu.usc.edu.cn; 4 School of Nursing, The Hong Kong Polytechnic University, Hong Kong, China; vickie.wang@polyu.edu.hk; 5 Joint Research Centre for Primary Health Care, The Hong Kong Polytechnic University, Hong Kong, China

**Keywords:** Patient-Reported Outcomes, stroke patients, functional status, quality of life, chronic conditions

## Abstract

**Background/Objectives:** Stroke is the leading cause of death and disability among adults in China. Most survivors experience debilitating symptoms, including limb weakness, pain, fatigue, and depression, which impair quality of life and increase caregiver burden. This study aimed to identify clusters of symptoms and functional status in patients with stroke, their associated factors, and the influence of family burden. **Methods:** From February 2023 to February 2024, 779 patients with stroke from three Chinese tertiary hospitals completed an electronic questionnaire covering demographics and Patient-Reported Outcomes Measurement Information System measures, including depression, anxiety, pain interference, physical and cognitive function, social participation, and family burden. Using SPSS 26.0 and Mplus 8.0, chi-square tests, analysis of variance, latent class analysis, and multiple logistic regression were performed. **Results:** Latent class analysis identified three distinct symptom–functional clusters among stroke patients: “low-symptom high-function” (36.4%), “moderate-symptom moderate-function” (54.6%), and “high-symptom low-function” (9.0%). Multinomial logistic regression revealed that age ≥ 60 years (OR = 0.15, *p* = 0.001), freelancing (OR = 2.75, *p* = 0.019), unemployment (OR = 2.73, *p* = 0.018), monthly income ≥ 5000 yuan (OR = 2.56, *p* = 0.026), no medical insurance (OR = 4.26, *p* = 0.01), living alone (OR = 2.45, *p* = 0.031), and family burden (OR = 0.95, *p* < 0.001) were associated with the “high-symptom low-function class”. **Conclusions:** Chinese stroke patients exhibit substantial heterogeneity in symptom and functional profiles. To optimize stroke rehabilitation, healthcare professionals should develop targeted management strategies, prioritizing high-risk populations, particularly socio-economically vulnerable and socially isolated patients.

## 1. Introduction

Stroke has become the leading cause of death and disability among Chinese adults [1]. In 2019, the incidence rate of stroke in China was 276.7 per 100,000 population [2]. Due to the disease itself and its treatment, approximately 70–80% of patients with stroke require prolonged recovery after acute treatment, experiencing symptoms such as limb weakness, pain, fatigue, and depression [3]. Post-stroke depression (PSD) can negatively affect recovery, leading to poorer quality of life (QOL), an increased risk of cognitive impairment, reduced social participation, and a higher risk of all-cause mortality [4,5,6,7]. Post-stroke fatigue (PSF) can lead to decreased functional ability, increased dependence on activities of daily living, reduced QOL, and increased mortality [8]. Nevertheless, individuals with post-stroke pain (PSP) frequently experience increased fatigue, depression, cognitive decline, functional impairment, and lower QOL [9].

Stroke affects not only a single domain of health but also multiple domains, including depression, fatigue, pain, cognition, and functional status [10]. Patients with Stroke experience multidimensional symptoms and symptom burden within 1 year after stroke onset [3]. According to the literature, one-third of patients with stroke experience PSD, and 44.19% experience post-stroke anxiety [6,11]. In addition, patients with stroke may also experience PSF [12]. Moreover, PSP is a common complication after stroke and is associated with depression, cognitive dysfunction, and impaired QOL [9]. Approximately 55–75% of patients with stroke have varying degrees of upper limb motor dysfunction [1]. Clinical manifestations predominantly present as a flexor pattern characterized by shoulder adduction and internal rotation, elbow flexion, and, in some patients, forearm pronation and wrist and finger flexion. Hefter et al. [13] defined five characteristic arm spasticity patterns (ASP I–V) based on the positions of the shoulder, elbow, forearm, and wrist joints. In a validation study of 665 post-stroke patients, 94% of spastic arm postures could be classified into one of these five patterns. The most frequent pattern was ASP III (41.8%), characterized by internal rotation and adduction of the shoulder with elbow flexion, coupled with neutral positioning of the forearm and wrist [13]. Additionally, more than 33.33% of patients are still unable to walk independently 3 months after discharge [14].

Literature has shown that patients with recent stroke exhibit distinct clinical profiles, which may result from differences in health-related quality of life (hrQoL) [10]. Katzan, Schuster, Bain, and Lapin [10] identified five distinct categories of post-stroke symptoms, including depression, pain, fatigue, and cognitive impairment, which are associated with differences in hrQoL. According to Shi, Li, Yang, Liu, and Xia [3], patients with stroke experience an average of 12 symptoms within 1 year after stroke, including limb weakness, inability to perform self-care, uncoordinated movements, impaired balance, fatigue, memory decline, shoulder pain, slow responses, and unclear speech [15]. Another study also explored three latent classes of psychoneurological symptoms in patients with acute stroke and helped identify subgroups with different symptom experiences. Although many studies have identified different symptom categories in patients with stroke, these studies primarily focus on the impact of symptoms on QOL or activities of daily living, as well as the relationship between symptoms and functional outcomes. There is still a lack of evidence regarding symptom clusters related to PSD, fatigue, and pain using the Patient-Reported Outcomes Measurement Information System (PROMIS). Post-stroke symptoms and functional outcomes are dynamically interrelated. Guided by the biopsychosocial model, this study conceptualized depression, fatigue, pain interference, physical function, cognitive function, and social participation as interconnected components of recovery.

Previous research has found that low socioeconomic status, including lower educational level, female sex, and lower income, is associated with PSD, functional impairment, PSF, and PSP [16,17,18,19]. Additionally, employment status [3] and body mass index (BMI) [20,21] may influence post-stroke symptoms in patients with stroke.

As patients with stroke experience diverse symptoms, their needs vary widely, ranging from ambulation and communication support to nursing care and emotional and psychological adjustment to the consequences of stroke, resulting in a substantial family caregiver burden [22]. Caregivers report a moderate to extreme physical and economic burden associated with caring for patients with stroke [23]. Low household income, female sex, caregiving for more than three months, and providing care for more than six hours per day are all strongly associated with the responsibilities of being primary caregivers for stroke survivors [24]. At present, few studies have focused on the symptoms, functional status, and family burden of patients with stroke in China and their associated factors. Therefore, this study aims to identify distinct symptom–function subtypes among patients with stroke, to examine the demographic and socioeconomic factors associated with each subtype and to explore the impact of family burden on subtype classification, with the ultimate goal of providing a scientific basis for clinical healthcare professionals to develop precise, individualized, and stratified nursing and rehabilitation intervention strategies tailored to patients with different subtypes.

## 2. Methods

### 2.1. Study Design

**Figure 1:**
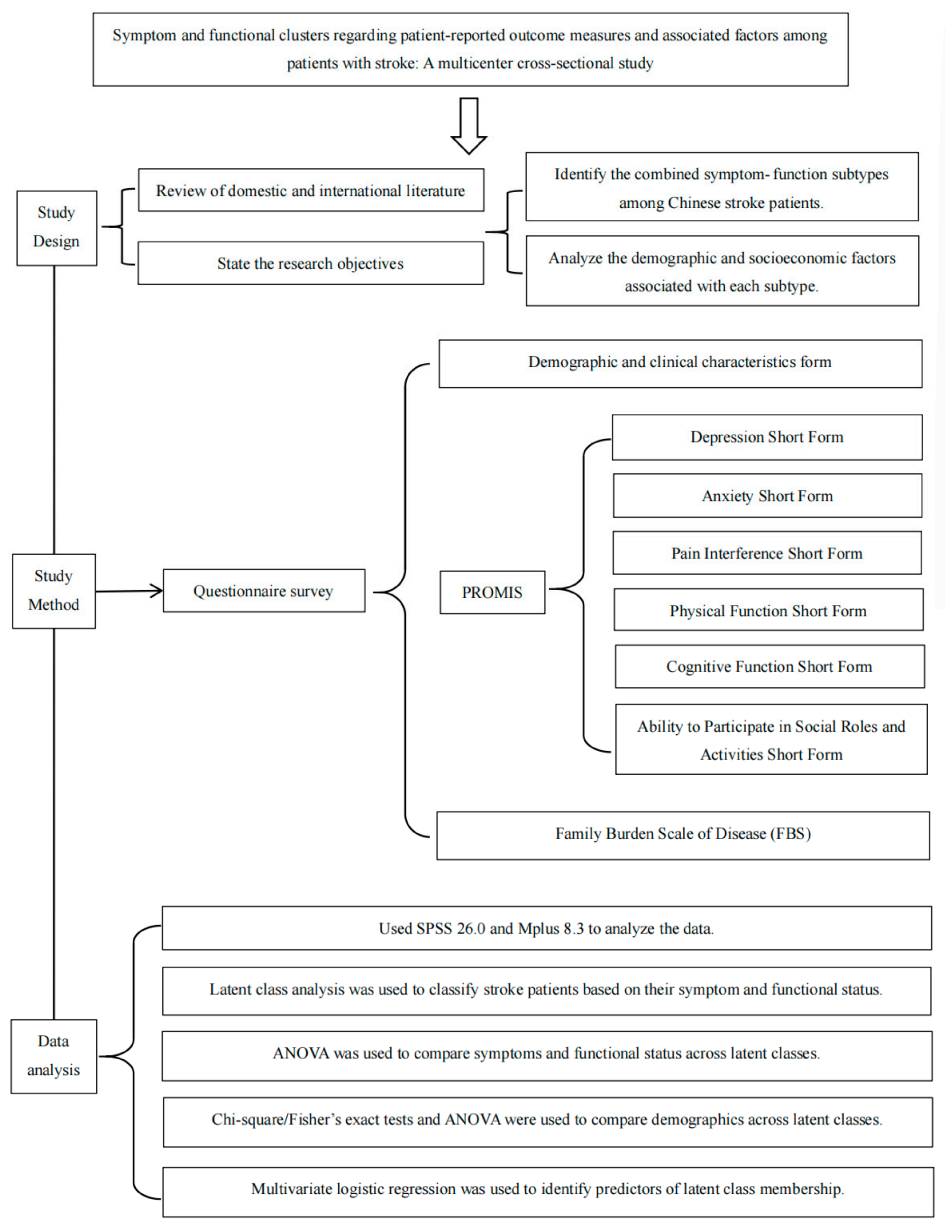
Study design and methods flowchart.

The present study employed a cross-sectional, multicenter design to identify latent classes of symptom and functional status in patients with stroke, as well as their associated factors and family burden. It was conducted between February 2023 and February 2024. This study is reported following the Chinese version of the Strengthening the Reporting of Observational Studies in Epidemiology (STROBE) checklist for cross-sectional studies. The study flowchart is shown in Figure 1.

### 2.2. Study Setting and Sampling

Using convenience sampling, 779 Chinese patients with stroke were recruited from tertiary hospitals in the provinces of Hunan, Zhejiang, and Hainan, China. The participants were patients who were followed up after hospital discharge. The average duration since stroke diagnosis was 13 months (range: 0.5–39 months). The inclusion criteria were: (1) age ≥ 18 years; (2) a confirmed diagnosis of stroke; (3) clear consciousness and no communication barriers; and (4) adequate reading ability and the capacity to understand health education materials and complete structured questionnaires. Patients with severe cardiac, hepatic, or renal dysfunction, as well as those with a history of malignant tumors or psychiatric disorders, were excluded.

### 2.3. Measurements

The instruments used for data collection included a demographic and clinical characteristics form and the Chinese version of the PROMIS forms. The PROMIS National Center in China provided usage licenses for the PROMIS instruments.

#### 2.3.1. Demographic and Clinical Characteristics Form

The demographic and clinical characteristics form was developed by the researchers and comprised gender, age, marital status, education level, employment status, place of residence, monthly income, medical insurance status, and BMI. The monthly income grouping in this study was determined based on the resident income levels published by the National Bureau of Statistics [25], with 5000 yuan used as the threshold for medium household monthly income.

#### 2.3.2. PROMIS-Depression Short Form

The Chinese version of the PROMIS Depression Short Form developed by Huang, Wu, Zhang, Tian, Huang, Huang, Zhou, He, and Wang [26] was used to assess depressive symptoms. Respondents rated their depression frequency over the past 7 days on a 5-point Likert scale (never, rarely, sometimes, often, and always). Total scores range from 8 to 40, with higher scores indicating more severe depression. The raw scores were converted to standardized T-scores (mean = 50, SD = 10). Cronbach’s alpha for this scale was 0.91.

#### 2.3.3. PROMIS-Anxiety Short Form

The Chinese version of the 8-item PROMIS Anxiety Short Form [26] was used to assess anxiety symptoms. The scale is a 5-point Likert-type scale ranging from 1 (never) to 5 (always), with a 7-day recall period. A total raw score of 8 indicates minimal anxiety, whereas a score of 40 indicates extreme anxiety. Item scores are summed to obtain the total score. Standardized T-scores were then generated from the raw scores (mean = 50, SD = 10). Higher scores indicate greater levels of anxiety.

#### 2.3.4. PROMIS-Pain Interference Short Form

Pain interference was measured using the Chinese version of the 6-item PROMIS Pain Interference Short Form [26]. Responses were scored on a 5-point Likert-type scale ranging from 1 (never) to 5 (always), with a 7-day recall period. Raw scores were summed, yielding a total score ranging from 6 (no interference) to 30 (severe interference). These raw scores were then converted into standardized T-scores (mean = 50, SD = 10), with higher scores indicating greater pain interference. The internal consistency of the scale was 0.90.

#### 2.3.5. PROMIS-Physical Function Short Form

The Chinese version of the 8-item PROMIS Physical Function Short Form [26] was used to assess physical function. Unlike the symptom-related subscales, this scale is scored such that total raw scores range from 8 (unrestricted physical function) to 40 (severely restricted physical function). Raw scores were then converted to standardized T-scores (mean = 50, SD = 10). Higher T-scores indicate better physical function. The Cronbach’s alpha coefficient for the scale was 0.95.

#### 2.3.6. PROMIS-Cognitive Function Short Form

For the assessment of cognitive function, the Chinese version of the 4-item PROMIS Cognitive Function Short Form [26] was used. Similar to the aforementioned physical function short form, this scale assesses cognitive function within the past 7 days, with a minimum raw score of 4 (unrestricted cognitive function) and a maximum score of 20 (severely restricted cognitive function). Raw scores were converted to standardized T-scores (mean = 50, SD = 10). Higher T-scores indicate better cognitive function. The Cronbach’s alpha coefficient for this scale was 0.97.

#### 2.3.7. PROMIS-Ability to Participate in Social Roles and Activities Short Form

To assess social function in patients with stroke, we used the Chinese version of the 4-item PROMIS Ability to Participate in Social Roles and Activities Short Form [26]. The scoring method is identical to that of the cognitive function scale described above, with a minimum raw score of 4 (unrestricted ability to participate in social roles and activities) and a maximum score of 20 (severely restricted ability to participate in social roles and activities). Raw scores were converted to standardized T-scores (mean = 50, SD = 10). Higher scores indicate better social function. The Cronbach’s alpha coefficient for this scale was 0.97.

#### 2.3.8. Family Burden Scale of Disease (FBS)

Family burden was assessed using the Chinese version of the FBS [27]. The scale includes 24 items across six dimensions: financial burden (6 items), impact on family routine (5 items), impact on family leisure (4 items), impact on family interaction (5 items), impact on the physical health of other family members (2 items), and impact on the mental health of other family members (2 items). The scale is rated using a 3-point Likert scale (0–2), where 0 indicates no burden, 1 indicates moderate burden, and 2 indicates severe burden. Higher scores indicate a greater burden. Due to the unequal number of items across dimensions, scores were standardized following the method of Lu et al., whereby scores for each dimension were divided by the number of items in that dimension to obtain standardized dimension scores. Similarly, a standardized total score was calculated. Using the mean of these standardized scores as cut-off values, scores were categorized into three levels and assigned values of 0, 1, and 2, respectively. The Cronbach’s alpha coefficient for the overall scale in this study was 0.935, and the Cronbach’s alpha coefficients for the individual dimensions ranged from 0.73 to 0.87.

### 2.4. Data Collection

Data were collected using an online questionnaire. The researchers utilized the online assessment tool “Questionnaire Star,” which is accessible on smartphones and integrated into the WeChat platform. A cover letter summarizing the study objectives was provided to all participants, and a QR code embedded in the survey instrument was distributed through WeChat groups for participants to scan and complete the questionnaire on their mobile devices, with an estimated completion time of 20 to 30 min. To ensure data quality, duplicate responses and questionnaires completed too quickly (<1 min) or too slowly (>1 h) were excluded from analysis. Incomplete questionnaires with missing data were also excluded. A total of 779 patients with stroke provided valid responses and were included in the final analysis. Due to the nature of the study, participants and study personnel could not be blinded; however, data collection and data analysis were conducted by different research teams, and the personnel responsible for data analysis did not participate in survey administration. All collected data were managed using EpiData (version 3.1), and data analysis was not initiated until the research team confirmed dataset accuracy and completeness.

### 2.5. Data Analysis

This study used SPSS 26.0 and Mplus 8.3 for data analysis. All eligible data were included in the analysis to ensure data integrity. Categorical variables were presented as frequencies and percentages, whereas continuous variables were described as mean, median, range, and standard deviation. Latent class analysis (LCA) was used to classify symptom and functional status in patients with stroke. The LCA model was estimated under the assumption of local independence, which posits that within each latent class, observed indicator variables are conditionally independent of one another. Bayesian information criterion (BIC), Akaike information criterion (AIC), and sample-size adjusted Akaike information criterion were used for model selection. A better model fit is indicated by lower values of these indices. When both the likelihood ratio test (Lo–Mendell–Rubin test [LMRT]) and bootstrapped likelihood ratio test (BLRT) are statistically significant (*p* < 0.05), the k-class model is considered superior to the k − 1 class model.

The chi-square test or Fisher’s exact test and ANOVA were used to examine differences in demographic characteristics across latent classes. In addition, one-way ANOVA was used to compare differences in symptom severity and functional status across latent classes. Multivariate logistic regression analysis was performed to identify predictors of latent classes of symptom and functional status. Logistic regression models were constructed based on results from the chi-square test and ANOVA. Latent class membership was treated as the dependent variable, demographic variables as independent variables, and the “low-symptom high-function class” as the reference category.

### 2.6. Ethical Considerations

Ethical approval was obtained from Fudan University (approval number: 241290-34) and the University of South China (approval number: 202314057822). All data were treated confidentially and anonymized. All information was processed in a confidential manner. All participating patients with stroke provided written informed consent prior to enrollment in the study.

## 3. Results

### 3.1. Demographic and Clinical Characteristics

A total of 779 patients with stroke participated in the study. The mean family burden score was 20.74 (SD = 11.6). The majority of participants were aged ≥ 60 years (68.4%), BMI category was 18.5–24.0 kg/m^2^ (48.1%), More than half of the participants were male (65.3%), were diagnosed with ischemic stroke (60.6%), were married (86.4%), had a primary school education or below (50.1%), were retired (40.4%), lived in urban or semi-urban areas (59.4%), had a monthly income below 5000 yuan (83.1%), had resident medical insurance (63.7%), and did not live alone (86.4%) (Table 1).

### 3.2. LCA

We iteratively fitted 1–4 models using symptoms of depression, fatigue, and pain interference, and functional indicators including physical function, cognitive function, and ability to participate in social roles and activities. The model fit indices for the latent class solutions are summarized in Table 2. The AIC, BIC, and adjusted BIC (aBIC) values were lowest for the three-class model. In addition, both the LMRT and the bootstrap likelihood ratio test (BLRT) indicated that the three-class solution was significantly better than the two-class model (*p* < 0.05). However, when a fourth class was added, the BLRT was not significant, suggesting no improvement over the three-class solution. Entropy values across all models ranged from 0.60 to 0.72, indicating moderate classification uncertainty. Therefore, the three-class solution was selected as the optimal model, capturing the main heterogeneity patterns in the data, although some overlap between adjacent classes was observed.

**Figure 2:**
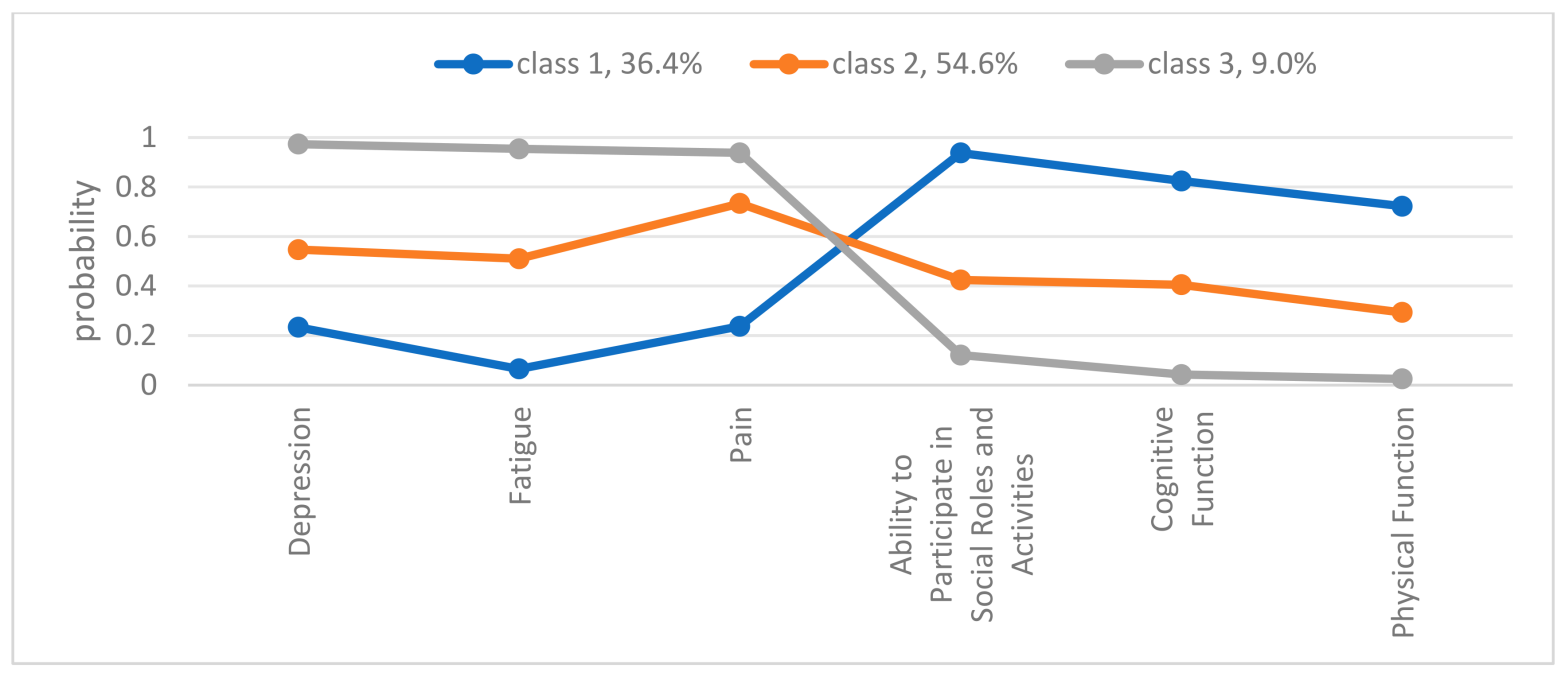
Probability of symptoms and functions classes.

The conditional probability distributions of the three latent classes of patients with stroke across three symptom domains and three functional domains are shown in Figure 2. The three latent classes were defined based on their characteristic profiles across different indicators. The blue line was labeled Class 1, the “low-symptom high-function class,” as it was characterized by a low probability of symptom endorsement and a high probability of preserved function. The orange line was classified as Class 2, the “moderate symptom and function class.” Class 3 (grey line) was labeled the “high-symptom low-function class.”

### 3.3. Differences in Symptoms and Functional Status Across Latent Classes

Raw scores were converted to standardized T-scores. The PROMIS scale used in this study has a mean T-score of 50 and a standard deviation of 10. A T-score of 40–60 indicates performance at the population baseline level. Scores between 30 and 40 indicate below-average performance relative to the general population, whereas scores below 30 indicate significantly below-average performance. Conversely, a T-score of 60–70 indicates above-average performance, while scores greater than 70 indicate significantly above-average performance.

The results showed significant differences in symptom and functional scores among the three latent classes. The symptom scores of the “high-symptom low-function class” were higher than the population baseline level, whereas functional scores were below the baseline level. Depression symptoms in the “low-symptom high-function class” were higher than the baseline level, while other symptoms and functional indicators were at baseline. Depression and pain symptoms in the “moderate symptom and function class” were higher than the baseline level, whereas fatigue symptoms and functional indicators were at baseline (Table 3).

### 3.4. Factors Associated with Latent Classes

As shown in Table 4, chi-square tests and ANOVA indicated significant differences in age (*p* = 0.01), BMI (*p* = 0.01), diagnosis (*p* = 0.013), marital status (*p* < 0.001), employment status (*p* = 0.003), monthly income (*p* = 0.010), medical insurance status (*p* < 0.001), residential status (*p* = 0.001), and family burden (*p* < 0.001) among the three latent classes defined by symptom and functional profiles. Variables that were significant in the chi-square tests and ANOVA were entered into the logistic regression model. The variable assignments used in the multivariate analysis are presented in Table 5.

The logistic regression results indicated that age ≥ 60 years (OR = 4.73, *p* = 0.005), ischemic stroke (OR = 1.62, *p* = 0.004), freelancing (OR = 2.08, *p* = 0.01), monthly income ≥ 5000 yuan (OR = 1.64, *p* = 0.013), resident medical insurance (OR = 0.42, *p* < 0.001), living alone (OR = 2.33, *p* = 0.002), and family burden (OR = 0.95, *p* < 0.001) were associated with membership in Class 2, the “moderate symptom and function class.” Compared with the “low-symptom high-function class,” age ≥ 60 years (OR = 0.15, *p* = 0.001), freelancing (OR = 2.75, *p* = 0.019), unemployment (OR = 2.73, *p* = 0.018), monthly income ≥ 5000 yuan (OR = 2.56, *p* = 0.026), no medical insurance (OR = 4.26, *p* = 0.01), living alone (OR = 2.45, *p* = 0.031), and family burden (OR = 0.95, *p* < 0.001) were associated with Class 3, the “high-symptom low-function class” (Table 6).

## 4. Discussion

### 4.1. Profile of Symptoms and Functional Status of Patients with Stroke

The current study identified three latent classes of symptoms and functional status in patients with stroke, including Class 1, the “low-symptom high-function class,” Class 2, the “moderate symptom and function class,” and Class 3, the “high-symptom low-function class.” Among these, patients with moderate symptoms and functional impairment accounted for the majority (54.6%), followed by those with low symptoms and high function (36.4%). In these two classes, most cases involved ischemic stroke. However, compared with earlier studies, the number and types of symptom classes differed significantly in this study. Katzan, Schuster, Bain, and Lapin [10] identified five latent symptom classes in patients with stroke based on hrQoL and patient-reported outcome measures, which were tentatively categorized as “excellent hrQoL,” “disabled with mixed hrQoL,” “mild limitations with average hrQoL,” “mild limitations with poor hrQoL,” and “disabled with poor hrQoL.” Another LCA classified patients with acute stroke with psychoneurological symptoms into “all high symptoms,” “high psychological symptoms,” and “all low symptoms” [15]. Variations in study populations, clinical and demographic characteristics, inclusion and exclusion criteria, disease profiles, and statistical methods may all contribute to discrepancies between studies.

### 4.2. Differences in Symptom and Functional Scores Among Different Latent Classes

This study found that depression, fatigue, and pain symptoms were higher in the “high-symptom low-function class” than in the other two classes. These results suggest that this pattern may be more prevalent among patients with a higher symptom burden. The findings are consistent with previous studies in China [15] and further corroborate the “disabled with poor hrQoL” phenotype proposed by Krishnagopal et al. [28], suggesting that the “high-symptom/low-function” pattern demonstrates cross-population stability across different stroke patient groups. Moreover, this phenotype has shown consistency across different care settings [29] and assessment modalities [30].

The present study also found that depression scores were above baseline in all three latent classes. Previous research has suggested that emotional disturbances may occur during the post-stroke phase due to the sudden onset of the disease and the severity of post-stroke sequelae [31]. The mean age of patients across the three classes was over 60 years, and PSD is more common among older patients [31,32]. Functional scores in the “high-symptom low-function class” were below baseline and significantly lower than those in the other two classes. This indicates that patients with severe post-stroke symptoms often experience impairments in social, cognitive, and physical functioning. These findings are consistent with previous studies [33,34]. Overall, these results highlight the need for clinicians to focus on symptom management in patients with severe post-stroke conditions, as symptom burden may co-occur with impairments in social, cognitive, and physical function.

### 4.3. Factors Associated with Latent Classes

The current study found that older patients (≥60 years) were more likely to belong to the “moderate symptom and function class.” Older patients experienced more severe PSD, fatigue, pain, and functional disability than younger patients, which is generally consistent with previous reports indicating that neurological deficits and functional disabilities tend to be more severe among older patients with stroke [35,36]. The evidence suggests that, after stroke, survivors experience moderate-to-severe functional impairments, and that these limitations worsen with aging more rapidly than in the general population [37].

In addition, patients with stroke engaged in freelancing work were more likely to belong to the “high-symptom low-function class” as well as the “moderate symptom and function class,” suggesting that individuals in these classes may be more vulnerable to work- and life-related stress. This vulnerability may be associated with more severe physical and mental health symptoms. These patients may be more likely to neglect physical health concerns, leading to ineffective control of risk factors and delayed monitoring and management of symptoms [38]. Furthermore, patients with stroke with unemployment status were more likely to belong to the “high-symptom low-function class,” indicating that unemployment may be a risk factor for more severe post-stroke symptoms. This finding is consistent with a study conducted in Indonesia by Yamanie, Chalik Sjaaf, Felistia, Harry Susanto, Diana, Lamuri, and Miftahussurur [38], which reported that patients with stroke with unemployment and no insurance had more severe stroke outcomes.

Nevertheless, the current study found that a monthly income of ≥5000 yuan was a predictive factor for both the moderate symptom and function class and the high-symptom low-function class, indicating that higher income was associated with more severe symptoms, which is inconsistent with previous research conducted in the United States by Katzan, Thompson, Uchino, and Lapin [34], which found that lower household income was associated with poorer PROMIS outcomes across several domains, including depression, physical function, pain, and satisfaction with social roles. Several possible explanations may account for this contradictory finding. First, since all participants were recruited from urban tertiary hospitals, patients with higher income may have better access to healthcare and therefore seek medical attention at an earlier stage of disease, whereas those with lower income may present only when symptoms become more severe. This may lead to earlier symptom assessment among higher-income patients, paradoxically resulting in a higher reported symptom burden. Second, higher-income patients, due to better access to treatment, may survive longer and thus accumulate more treatment-related side effects over time. Third, higher-income individuals may have higher health expectations and therefore may report even mild symptoms as more severe discomfort, resulting in higher questionnaire scores.

Our study also revealed that patients with stroke with resident medical insurance, as well as those without medical insurance, were more likely to belong to the moderate symptom and function class and the high-symptom low-function class. The majority of patients in these classes shared a prominent demographic profile, including urban residence, self-employment or retirement status, and coverage under resident medical insurance. This finding may be explained by the fact that most participants were recruited from urban tertiary hospitals, where patients are predominantly residents living nearby. In addition, due to China’s implementation of an integrated basic medical insurance system for urban and rural residents, most patients with stroke are covered by resident medical insurance; however, its reimbursement rate is lower than that of employee-based medical insurance. Previous studies have shown that patients with employee medical insurance may have access to higher-quality medical resources [39,40]. However, the treatment costs associated with stroke are relatively high. According to the latest survey, the average hospitalization cost per ischemic stroke in China is 10,740.7 yuan [41].

According to the latest statistics, the per capita annual disposable income in Chinese urban areas is 51,821 yuan, while in rural areas it is 21,691 yuan [42]. For patients with stroke covered by resident medical insurance, such financial pressure can be substantial. Therefore, to cope with these cost burdens, patients may reduce healthcare utilization and opt for more cost-effective treatment and rehabilitation options. Compared with resident medical insurance, individuals with commercial insurance typically have more outpatient visits and longer hospital stays, suggesting that patients with stroke with commercial insurance may have greater access to medical resources, better treatment and rehabilitation experiences, and relatively milder symptoms [40]. According to our survey, most uninsured patients come from rural areas. Similar to freelancers, they tend to have lower health literacy, less attention to their health, and limited understanding of disease and may experience more severe psychological symptoms after disease onset [43].

Moreover, the current study also revealed that patients with stroke diagnosed with ischemic stroke were more likely to belong to the moderate symptom and function class, suggesting that the functional consequences of ischemic stroke may be relatively less severe than those of hemorrhagic stroke. According to disease characteristics, the mortality rate of hemorrhagic stroke is higher than that of ischemic stroke [44].

Additionally, this study found that patients with stroke living alone were more likely to belong to the “moderate symptom and function class” as well as the “high-symptom low-function class,” which may suggest that living with family could serve as a potentially protective factor for post-stroke symptoms and functional ability. Social support has been shown to buffer against depression in individuals without stroke. Depressive symptoms appear to be alleviated by emotional support from parents, spouses, family members, friends, relatives, and other significant others [45].

However, this study found that family burden was associated with both the “moderate symptom and function class” and the “high-symptom low-function class” among patients with stroke, indicating that higher family burden scores paradoxically corresponded to milder patient symptom profiles. This finding is inconsistent with previous research [46]. One possible explanation is that the scale was subjectively completed by caregivers. Family members providing long-term care for severely disabled patients may have become accustomed to high-care demands, resulting in relatively lower subjective burden scores; conversely, caregivers of patients with milder symptoms, experiencing a sudden disruption due to the stroke event, may report greater psychological contrast and higher perceived burden [47]. Additionally, the “high-symptom low-function class” had a small sample size, and caregivers of the most severely affected patients may have been unable to participate in the survey due to limited time and energy.

### 4.4. Strengths and Limitations

This study conducted a multicenter, large-sample cross-sectional survey, and the sample was representative to a certain extent; thus, generalizability is supported to some degree. In addition, this study identified characteristics of patients with stroke who may experience moderate symptoms and moderate function, as well as high symptoms and low function, following cerebrovascular events, which may facilitate the provision of personalized and precise care targeting potential symptoms and functional impairments in the early stages of the disease. However, several limitations should be acknowledged. First, the entropy value for the three-class solution was 0.67, indicating moderate classification accuracy and suggesting that discrimination between adjacent latent classes is imperfect, with a non-negligible risk of misclassification. Thus, while the three-class solution provides a useful heuristic framework for understanding sample-level heterogeneity, caution is warranted when applying it to individual-level clinical decision-making, and future studies are needed to further validate these subgroups. Second, the cross-sectional design precludes causal or temporal inference, as only statistical associations rather than causal relationships can be established. Third, the use of patient-reported outcome measures may introduce self-report bias, including recall bias and social desirability bias. Fourth, although several covariates were adjusted for, residual confounding due to unmeasured factors, such as social support, health literacy, and stroke severity, cannot be completely excluded. Fifth, key clinical variables, including stroke severity, lesion location, functional dependence, and rehabilitation exposure, were not collected, limiting the ability to fully account for their potential influence on latent class membership. Sixth, participants were predominantly recruited from urban tertiary hospitals, which may limit the generalizability of the findings to rural or community-based populations. Finally, only a cross-sectional design was used to explore factors associated with latent class membership; longitudinal studies are needed to validate the stability of these typologies and their associated determinants over time.

## 5. Conclusions

Stroke patients exhibit distinct heterogeneous patterns of post-stroke symptoms and functional status, with approximately 9.0% classified into the most vulnerable “high symptom and low function” profile. Multinomial logistic regression indicates that socioeconomic and demographic factors significantly shape these phenotypes. Specifically, earning a higher monthly income (≥5000 RMB), working in freelancing/unemployed sectors, without health insurance and living alone are key risk factors driving patients into groups with more severe clinical symptoms and poorer functioning. Conversely, advanced age (≥60 years) and family burden demonstrate potential protective associations against the highest symptom–burden class within this sample. These findings underscore the urgency of shifting from “one-size-fits-all” post-stroke care to tailored, multidimensional symptom management strategies that prioritize socio-economically vulnerable and socially isolated patients.

## Figures and Tables

**Table 1:** Baseline characteristics of the study participants (N = 779).

Variables	n (%)
Age (years)	
18–60	246 (31.6)
≥60	533 (68.4)
BMI (kg/m^2^)	
<18.5	41 (5.3)
18.5–24.0	375 (48.1)
24.0–28.0	279 (35.8)
≥28.0	84 (10.8)
Gender	
Male	509 (65.3)
Female	270 (34.7)
Diagnosis	
Hemorrhagic stroke	307 (39.4)
Ischemic stroke	472 (60.6)
Marital status	
Married	673 (86.4)
Single	53 (6.8)
Divorced	30 (3.9)
Widowed	23 (3.0)
Education level	
Primary school or below	390 (50.1)
High school and vocational school	315 (40.4)
College degree or above	74 (9.5)
Employment status	
Employed	182 (23.4)
Retired	315 (40.4)
Freelancing	151 (19.4)
Unemployed	131 (16.8)
Residence	
Urban	463 (59.4)
Rural	316 (40.6)
Monthly income (RMB)	
<5000	647 (83.1)
≥5000	132 (16.9)
Medical insurance	
Employee health insurance	144 (18.5)
Resident medical insurance	496 (63.7)
Commercial medical insurance	75 (9.6)
Without health insurance	64 (8.2)
Living arrangement	
Living alone	106 (13.6)
Not living alone	673 (86.4)
Family burdens (Mean ± SD)	20.74 ± 11.60

**Table 2:** Fit indices for the latent class analysis models.

Model	K	Log L	AIC	BIC	aBIC	Entropy	LMR-LRT (*p*)	BLRT (*p*)	Class Probability
1 Class	6	−3189.85	6391.69	6419.64	6400.59	—	—	—	1.000
2 Classes	13	−2940.45	5906.91	5967.46	5926.18	0.676	<0.001	<0.001	0.442, 0.558
3 Classes	20	−2928.69	5897.39	5990.55	5927.04	0.674	0.048	<0.001	0.364, 0.546, 0.090
4 Classes	27	−2923.90	5901.80	6027.56	5941.82	0.708	0.127	0.133	0.130, 0.250, 0.292, 0.328

Note. K: number of free parameters; Log L: Log-likelihood; AIC: Akaike Information Criterion; BIC: Bayesian Information Criterion; aBIC: Sample-size Adjusted BIC; LMR-LRT: Lo-Mendell-Rubin Likelihood Ratio Test; BLRT: Bootstrapped Likelihood Ratio Test.

**Table 3:** Comparison of clinical outcomes across the three latent classes (N = 779).

Variables	Class 1 (n = 297)	Class 2 (n = 411)	Class 3 (n = 71)	F	*p*
Depression	62.61 ± 10.90	64.27 ± 7.44	69.03 ± 13.62	25.92	<0.001
Fatigue	43.48 ± 8.90	57.72 ± 10.92	62.75 ± 6.71	219.66	<0.001
Pain interference	51.41 ± 13.00	62.05 ± 10.58	65.16 ± 7.34	90.25	<0.001
Ability to participate in social roles and activities	54.23 ± 10.54	43.01 ± 10.70	39.18 ± 8.51	120.86	<0.001
Cognitive function	51.57 ± 9.67	42.15 ± 9.81	38.83 ± 7.15	102.59	<0.001
Physical function	46.44 ± 11.75	36.03 ± 9.16	34.09 ± 9.50	101.02	<0.001

Note. Data are presented as Mean ± SD unless otherwise specified. F: F-statistic from analysis of variance (ANOVA).

**Table 4:** Comparison of baseline characteristics among the three latent classes (N = 779).

Variables	Class 1 (n = 297)	Class 2 (n = 411)	Class 3 (n = 71)	*p*
Age (years, Mean ± SD)	63.47 ± 11.91	65.47 ± 11.94	67.73 ± 12.17	0.010 ^a^
BMI (kg/m^2^)				0.010 ^b^
<18.5	8 (2.7)	27 (6.6)	6 (8.5)	
18.5–24.0	161 (54.2)	185 (45.0)	29 (40.8)	
24.0–28.0	103 (34.7)	153 (37.2)	23 (32.4)	
≥28.0	25 (8.4)	46 (11.2)	13 (18.3)	
Gender				0.593 ^b^
Male	195 (65.7)	264 (64.2)	50 (70.4)	
Female	102 (34.3)	147 (35.8)	21 (29.6)	
Diagnosis				0.013 ^b^
Hemorrhagic stroke	136 (45.8)	143 (34.8)	28 (39.4)	
Ischemic stroke	161 (54.2)	268 (65.2)	43 (60.6)	
Marital status				<0.001 ^b^
Married	273 (91.9)	344 (83.7)	56 (78.9)	
Single	19 (6.4)	28 (6.8)	6 (8.5)	
Divorced	0 (0.0)	28 (6.8)	2 (2.8)	
Widowed	5 (1.7)	11 (2.7)	7 (9.9)	
Education level				0.419 ^b^
Primary school or below	144 (48.5)	203 (49.4)	43 (60.6)	
High school and vocational school	126 (42.4)	166 (40.4)	23 (32.4)	
College degree or above	27 (9.1)	42 (10.2)	5 (7.0)	
Employment status				0.003 ^b^
Employed	88 (29.6)	82 (20.0)	12 (16.9)	
Retired	126 (42.4)	161 (39.2)	28 (39.4)	
Freelancing	40 (13.5)	96 (23.4)	15 (21.1)	
Unemployed	43 (14.5)	72 (17.5)	16 (22.5)	
Residence				0.277 ^b^
Urban	186 (62.6)	239 (58.2)	38 (53.5)	
Rural	111 (37.4)	172 (41.8)	33 (46.5)	
Monthly income (RMB)				0.010 ^b^
<5000	232 (78.1)	351 (85.4)	64 (90.1)	
≥5000	65 (21.9)	60 (14.6)	7 (9.9)	
Medical insurance				<0.001 ^b^
Employee health insurance	45 (15.2)	88 (21.4)	11 (15.5)	
Resident medical insurance	219 (73.7)	240 (58.4)	37 (52.1)	
Commercial medical insurance	12 (4.0)	57 (13.9)	6 (8.5)	
Without health insurance	21 (7.1)	26 (6.3)	17 (23.9)	
Living arrangement				0.001 ^b^
Not living alone	273 (91.9)	344 (83.7)	56 (78.9)	
Living alone	24 (8.1)	67 (16.3)	15 (21.1)	
Family burdens (Mean ± SD)	25.26 ± 11.95	18.03 ± 10.63	17.49 ± 9.42	<0.001 ^a^

Note. Data are presented as n (%) for categorical variables and Mean ± SD for continuous variables. ^a^ *p*-value calculated using analysis of variance (ANOVA); ^b^ *p*-value calculated using Chi-square test.

**Table 5:** Variable assignments and operational definitions for multivariate analysis.

Variables	Operational Definition and Assignment
Age	18–60 = 1; ≥60 = 2
BMI (kg/m^2^)	<18.5 = 1; 18.5–24.0 = 2; 24.0–27.9 = 3; ≥27.9 = 4
Diagnosis	Hemorrhagic stroke = 1; Ischemic stroke = 2
Employment status	Employed = 1; Retired = 2; Freelancing = 3; Unemployed = 4
Monthly income (RMB)	<5000 = 1; ≥5000 = 2
Medical insurance	Employee health insurance = 1; Resident medical insurance = 2; Commercial medical insurance = 3; Without health insurance = 4
Living arrangement	Not living alone = 1; Living alone = 2

**Table 6:** Multinomial logistic regression analysis of factors associated with latent class membership.

Variables	Moderate Symptom and Function Group			High Symptom Low Function Group		
	OR	95% CI	*p*	OR	95% CI	*p*
Age (years) ^a^						
≥60	4.73	1.59–14.08	0.005	0.15	0.05–0.48	0.001
BMI (kg/m^2^) ^b^						
18.5–24.0	0.49	0.21–1.16	0.105	0.31	0.09–1.06	0.063
24.0–27.9	0.74	0.31–1.78	0.504	0.46	0.13–1.66	0.238
≥27.9	1.23	0.46–3.32	0.676	1.18	0.28–4.97	0.819
Diagnosis ^c^						
Ischemic stroke	1.62	1.16–2.26	0.004	1.37	0.75–2.51	0.308
Employment status ^d^						
Retired	0.85	0.50–1.44	0.549	1.63	0.79–3.38	0.189
Freelancing	2.08	1.19–3.62	0.010	2.75	1.18–6.41	0.019
Unemployed	0.92	0.49–1.75	0.801	2.73	1.19–6.27	0.018
Monthly income (RMB) ^e^						
≥5000	1.64	1.11–2.42	0.013	2.56	1.12–5.86	0.026
Medical insurance ^f^						
Resident medical insurance	0.42	0.27–0.67	<0.001	0.67	0.29–1.56	0.355
Commercial medical insurance	1.63	0.75–3.56	0.219	1.73	0.45–6.59	0.425
Without health insurance	0.66	0.31–1.39	0.273	4.26	1.42–12.82	0.010
Living arrangement ^g^						
Living alone	2.33	1.37–3.97	0.002	2.45	1.09–5.53	0.031
Family burdens	0.95	0.93–0.96	<0.001	0.95	0.92–0.97	<0.001

Note. OR: odds ratio; CI: confidence interval. The reference class is the Low Symptom High Function Group. The reference categories for the independent variables are: ^a^ 18–60 years; ^b^ <18.5 kg/m^2^; ^c^ Hemorrhagic stroke; ^d^ Employed; ^e^ <5000 RMB; ^f^ Employee health insurance; ^g^ Not living alone.

## Data Availability

The data presented in this study are available on request from the corresponding author due to privacy reasons.
